# Classification of AIDS-related lymphoma cases between 1987 and 2012 in Japan based on the WHO classification of lymphomas, fourth edition

**DOI:** 10.1002/cam4.178

**Published:** 2014-01-10

**Authors:** Yasunori Ota, Tsunekazu Hishima, Makoto Mochizuki, Yoshinori Kodama, Suzuko Moritani, Naoki Oyaizu, Sohtaro Mine, Atsushi Ajisawa, Junko Tanuma, Tomoko Uehira, Shotaro Hagiwara, Keishiro Yajima, Yusuke Koizumi, Takuma Shirasaka, Yuki Kojima, Hirokazu Nagai, Yoshiyuki Yokomaku, Yumiko Shiozawa, Tomohiko Koibuchi, Aikichi Iwamoto, Shinichi Oka, Hideki Hasegawa, Seiji Okada, Harutaka Katano

**Affiliations:** 1 Department of Pathology and Laboratory Medicine, Institute of Medical Science, The University of TokyoShirokanedai 4-6-1, Minato-ku, Tokyo, 108-8639, Japan; 2 Department of Pathology, Tokyo Metropolitan Komagome HospitalHonkomagome 3-18-22, Bunkyo-ku, Tokyo, 113-8677, Japan; 3 Department of Pathology, National Center for Global Health and Medicine Hospital1-21-1 Toyama, Shinjuku-ku, Tokyo, 162-8655, Japan; 4 Department of Pathology, Kyorin University School of Medicine6-20-2 Shinkawa, Mitaka City, Tokyo, 181-8611, Japan; 5 Department of Pathology, Osaka National Hospital2-1-14 Hoenzaka, Chuo-ku, Osaka, 540-0006, Japan; 6 Department of Pathology, Nagoya Medical Center4-1-1 Sannomaru, Nakaku, Nagoya, 460-0001, Japan; 7 Department of Pathology, National Institute of Infectious Diseases1-23-1 Toyama, Shinjuku-ku, Tokyo, 162-8640, Japan; 8 Department of Infectious Diseases, Tokyo Metropolitan Komagome HospitalHonkomagome 3-18-22, Bunkyo-ku, Tokyo, 113-8677, Japan; 9 AIDS Clinical Center, National Center for Global Health and Medicine Hospital1-21-1 Toyama, Shinjuku-ku, Tokyo, 162-8655, Japan; 10 Department of Infectious Diseases, Osaka National Hospital2-1-14 Hoenzaka, Chuo-ku, Osaka, 540-0006, Japan; 11 Department of Hematology, National Center for Global Health and Medicine Hospital1-21-1 Toyama, Shinjuku-ku, Tokyo, 162-8655, Japan; 12 Department of Hematology, Clinical Research Center, Nagoya Medical Center4-1-1 Sannomaru, Nakaku, Nagoya, 460-0001, Japan; 13 Department of Infectious Diseases and Immunology, Clinical Research Center, Nagoya Medical Center4-1-1 Sannomaru, Nakaku, Nagoya, 460-0001, Japan; 14 Department of Infectious Diseases, Institute of Medical Science, The University of TokyoShirokanedai 4-6-1, Minato-ku, Tokyo, 108-8639, Japan; 15 Center for AIDS Research, Kumamoto UniversityKumamoto, 860-0811, Japan

**Keywords:** AIDS-related lymphoma, antiretroviral therapy, Burkitt lymphoma, diffuse large B-cell lymphoma, Epstein–Barr virus

## Abstract

The introduction of combined antiretroviral therapy (ART) has reduced the mortality of patients with human immunodeficiency virus-1 infection worldwide. However, malignant lymphoma is a severe and frequent complication seen in patients with acquired immunodeficiency syndrome (AIDS). The diagnostic criteria for some categories of AIDS-related lymphoma were revised in the World Health Organization International Classification of Lymphoma, fourth edition. The purpose of this study was to assess the clinicopathological characteristics of Japanese patients with AIDS-related lymphoma according to the revised classification. In this retrospective study, 207 AIDS-related lymphoma cases diagnosed between 1987 and 2012 in Japan were subjected to histological subtyping and clinicopathological analyses. Diffuse large B-cell lymphoma (DLBCL) was the predominant histological subtype throughout the study period (*n* = 104, 50%). Among the DLBCL cases, 24% were of the germinal center (GC) type and 76% were of the non-GC type. Non-GC-type cases showed a significantly lower 1-year survival rate (43%) than the GC-type cases (82%). Cases of Burkitt lymphoma (*n* = 57, 28%), plasmablastic lymphoma (*n* = 16, 8%), primary effusion lymphoma (*n* = 9, 4%), Hodgkin lymphoma (*n* = 8, 4%), and large B-cell lymphoma arising in Kaposi sarcoma-associated herpesvirus-associated multicentric Castleman disease (*n* = 2, 1%) were also observed. Hodgkin lymphoma was more common in patients receiving ART (11.1%) than in ART-naïve patients (1.4%). Statistical analyses identified CD10 negativity, BCL-6 negativity, Epstein–Barr virus positivity, and Kaposi sarcoma-associated herpesvirus positivity as risk factors for poor prognosis. This information will help in the early diagnosis of lymphoma in patients with AIDS.

## Introduction

Malignant lymphoma is a severe complication in patients with acquired immunodeficiency syndrome (AIDS). The incidence of lymphoma is 60- to 200-fold higher in patients with human immunodeficiency virus-1 (HIV-1) infection than in the general, uninfected patient population 1–3. AIDS-related lymphoma (ARL) has unique histological characteristics compared with lymphoma occurring in immunocompetent individuals. B-cell lineage lymphoma is a predominant subtype of ARL, whereas lymphomas involving other cell lineages, including T/natural killer (NK) cells, are very rare. Diffuse large B-cell lymphoma (DLBCL) is the most frequent histological subtype of ARL, and Burkitt lymphoma (BL) is another major subtype. Two oncogenic herpes viruses, Epstein–Barr virus (EBV) and Kaposi sarcoma-associated herpesvirus (KSHV or human herpesvirus 8), are also frequently associated with the pathogenesis of ARL 4. EBV positivity is often detected in cases of DLBCL, plasmablastic lymphoma (PBL), and primary effusion lymphoma (PEL). In addition, all PEL cases are positive for KSHV. PBL and PEL generally develop in patients with AIDS; they are very rare in immunocompetent hosts.

Antiretroviral therapy (ART) successfully and drastically reduces the HIV-1 RNA copy number in serum, resulting in recovery of immune function and decreased mortality in HIV-1-infected patients 5. ART has been shown to significantly decrease the incidence of opportunistic infections such as pneumocystis pneumonia, cytomegalovirus, and *Candida*. Although ART did not significantly reduce the incidence of lymphoma in HIV-1-infected individuals in previous studies 6–15, the clinicopathological characterization of ARL has changed because of the introduction of ART 16. Of note, the incidence of central nervous system (CNS) lymphomas has decreased in HIV-1-infected patients undergoing ART 16. However, the number of cases of BL and Hodgkin lymphoma (HL) have increased in patients undergoing ART 6,17,18. In particular, an increased number of HL cases have been reported in ART patients with high CD4 counts 18.

Few studies have presented the clinicopathological features of a large number of ARL patients in Asian countries 17,19,20. In Japan, more than half of ARL cases were categorized as EBV-associated DLBCL before the introduction of ART 17. However, after the introduction of ART, EBV positivity has decreased among DLBCL cases, and the incidence of BL cases has increased among AIDS patients. In addition, the frequency of nodal involvement in lymphoma cases has increased in patients undergoing ART, whereas the incidence of CNS lymphomas has decreased 17. Thus, ART induction has altered the clinicopathological characteristics of ARL in Japan.

In 2008, the World Health Organization (WHO) released the fourth edition of the classification of lymphomas based on recent accumulation of scientific evidence for these diseases 21. Some definitions of lymphomas observed frequently in HIV-1-infected patients were altered in the revised classification 22. For example, some variants, subgroups, and subtypes of DLBCL were redefined 23. The definition of atypical Burkitt/Burkitt-like variant was excluded from the chapter on BL 24, and the definition of B-cell lymphoma, unclassifiable, with features intermediate between DLBCL and BL (intermediate DLBCL/BL) was newly added 25. PBL and PEL were explained in more detail, and the definition of another KSHV-positive lymphoma, large B-cell lymphoma arising in patients with KSHV-associated multicentric Castleman disease, was added 26,27. Thus, the revised WHO classification of lymphomas is likely to affect the histological classification of ARL cases. However, thus far, few reports have described the histological classification of lymphoma in HIV-1-infected patients according to this edition of the WHO classification.

In the present study, ARL cases were classified according to the fourth edition of the WHO classification of lymphomas, and alterations in the clinicopathological characteristics of ARL due to the introduction of ART were investigated. In addition, the correlations of some biomarkers with the prognosis of ARL are discussed.

## Materials and Methods

### Patients

Studies using human tissue were performed with the approval of the Institutional Review Boards of the National Institute of Infectious Diseases (Approval No. 344) and of five hospitals in Japan: Tokyo Metropolitan Komagome Hospital; National Center for Global Health and Medicine Hospital; the Institute of Medical Science, the University of Tokyo; Osaka Medical Center; and Nagoya Medical Center Hospital. The clinical data of 207 cases of ARL, diagnosed histologically between January 1987 and November 2012, were investigated retrospectively (Table 1). These patients were referred to one of the five hospitals mentioned above. As 325 cases of ARL were reported in all of Japan during the study period, according to a national survey on the clinical manifestation of patients with HIV-1 infection (Yasuoka A. 2012 Annual report of the Health and Labor Sciences Research Grants for AIDS from the Ministry of Health, Labor and Welfare Japan, Japanese), we could safely assume that approximately two-thirds of all ARL cases in Japan were covered in the present study. The clinical data, such as age, gender, risk factors, CD4 cell count, use of ART, and prognosis, were collected from the medical records of the various hospitals. The CD4 cell counts at the time of lymphoma diagnosis were considered. In this study, ART was defined as the prescription of at least one antiretroviral drug, including a protease inhibitor or a nonnucleoside reverse transcriptase inhibitor. In Japan, ART was introduced in 1996, and the first lymphoma case of patient on ART appeared in 1997. Thus, we divided the patients into two groups based on their date of diagnosis: the pre-ART era group (*n* = 43), including those diagnosed from 1987 to before the first lymphoma case of the ART (+) patient in 1997, and the ART era group (*n* = 164), including all other cases diagnosed from 1997 to 2012. ART status was not available for seven patients in the ART era group. CNS and lymph node (LN) involvement of the lymphoma were determined according to autopsy records or clinical records.

**Table 1:** Characteristics of patients diagnosed with AIDS-related lymphoma.

Factor	Total	DLBCL	BL	PBL	PEL	HL	LBL-KSHV-MCD	Other
*n*	207	104	57	16	9	8	2	11
Age, years (mean) [median, range]	45.4 [44, 12–76]	45.7 [44, 12–76]	43.7 [41, 25–59]	47.9 [51, 31–59]	43.5 [43, 30–59]	53.5 [56, 39–65]	48.5 [49, 39–65]	42.1 [40, 25–59]
Men (%)	198 (96)	97 (93)	55 (97)	16 (100)	9 (100)	8 (100)	2 (100)	11 (100)
CD4 (mean) [median, range]	149 [82, 0–2413]	86 [41, 0–824]	249 [216, 14–652]	85 [59, 7–394]	75 [20, 6–260]	235 [236, 22–497]	206 [206, 30–382]	303 [70, 2–2431]
ART (+) at onset (%)	26.1	22.1	31.6	18.8	11.1	75.0	50.0	18.2
EBV-positive (%)	59.9	70.3	27.3	93.8	66.7	100.0	0.0	54.5
CNS involvement (%)	29.6	43.8	20.0	9.1	0.0	0.0	0.0	20.0
LN involvement (%)	44.3	30.5	50.9	58.3	55.6	100.0	50.0	54.5
BM involvement (%)	30.1	11.4	47.2	33.3	50.0	71.4	0.0	45.5
1-year survival rate (%)	52.2	42.3	68.4	62.5	33.3	75.0	0.0	54.5

ART, antiretroviral therapy; BM, bone marrow; BL, Burkitt lymphoma; CNS, central nervous system; DLBCL, diffuse large B-cell lymphoma; EBV, Epstein–Barr virus; HL, Hodgkin lymphoma; LBL-KSHV-MCD, large B-cell lymphoma arising in Kaposi sarcoma-associated herpesvirus (KSHV)-associated multicentric Castleman disease; LN, lymph node; PBL, plasmablastic lymphoma; PEL, primary effusion lymphoma.

### Immunohistochemistry and in situ hybridization

The cell lineage of each case was determined using immunohistochemistry, as described previously 28. CD3, CD10, CD20, CD30, CD38, CD45RO, CD79a, CD138, BCL-2, BCL-6, IRF4/MUM1, cIgM, immunogloblin light-chain lambda, kappa, Ki67 (MIB-1), LMP-1, and EBNA-2 antibodies were used as primary antibodies. The presence of EBV was examined using in situ hybridization for EBV-encoded small RNAs (EBER), as described previously 29. KSHV was detected by immunohistochemistry using an antibody against KSHV-encoded latency-associated nuclear antigen 1 (LANA-1) 30. *MYC* rearrangement was investigated using fluorescent in situ hybridization on paraffin sections, as described previously 31.

### Subtyping of lymphomas

**Figure 1:**
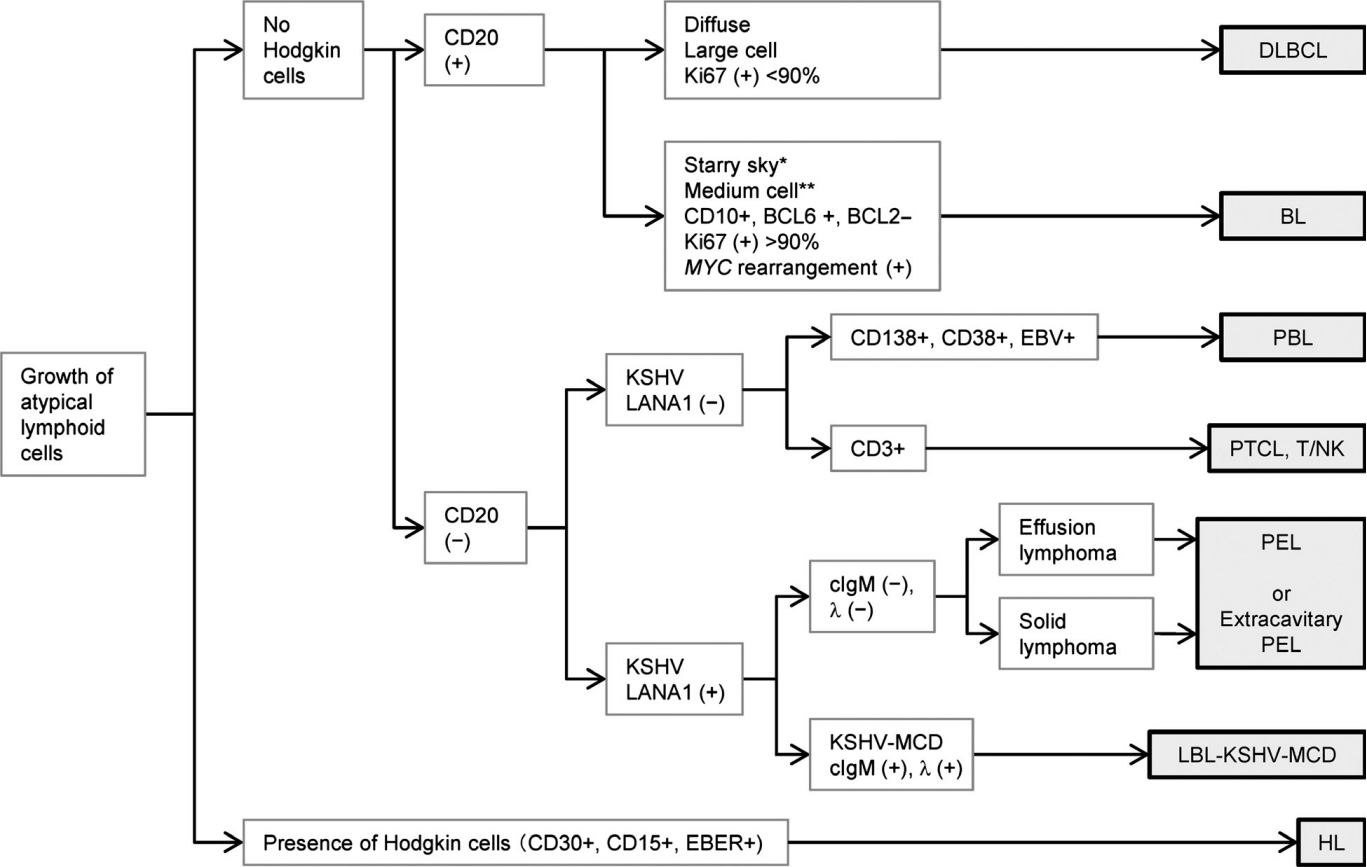
Diagnostic flowchart for AIDS-related lymphoma. CD20-positive cases were categorized as diffuse large B-cell lymphoma or Burkitt lymphoma (BL) according to their morphology, immunophenotype, and *MYC* rearrangement. Some BL cases did not show the typical morphology of BL, such as the starry sky pattern (*) and medium-sized cells (**). For the CD20-negative group, if positive for Kaposi sarcoma-associated herpesvirus (KSHV)-encoded latency-associated nuclear antigen 1 (LANA-1), the case was categorized as primary effusion lymphoma or large B-cell lymphoma arising in KSHV-associated multicentric Castleman disease. KSHV-negative cases were examined using immunohistochemistry for CD3, CD138, CD38, and in situ hybridization for EBV to determine its subtype (see Table 1 for abbreviations).

The histological subtyping of lymphomas was based on the fourth edition of the WHO classification 21. All cases were reviewed by five pathologists (YO, TH, MM, YK, and HK) and classified according to a flowchart (Fig. 1). The diagnosis of BL was based on histological, immunohistochemical, and chromosomal data, as recommended in the revised WHO classification system. DLBCL was subclassified into the germinal center (GC) type and the non-GC type, according to the algorithm reported previously by Hans et al. 32.

### Statistical analyses

Analyses of statistical significance were performed using the chi-square test for bivariate tabular analysis and using the Mann–Whitney test for comparison of two independent groups of sampled data, such as the CD4 cell count.

## Results

### Histological classification of ARL

**Figure 2:**
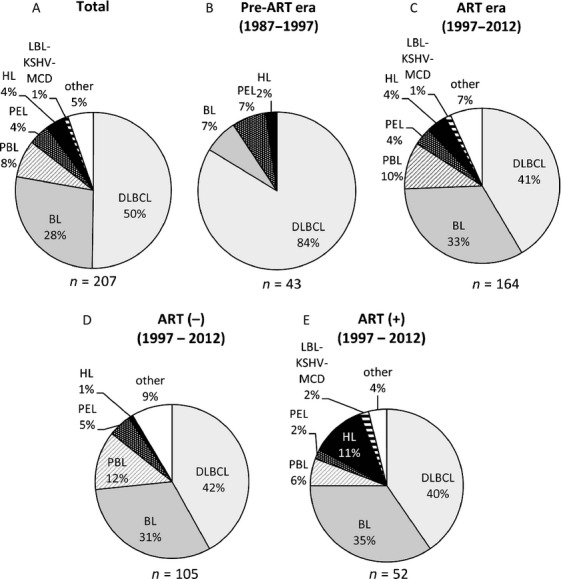
Pie charts for the histological subtype of AIDS-related lymphomas. The histological subtypes of AIDS-related lymphomas (ARLs) during the entire study period (1987–2012, panel A), the preantiretroviral therapy (pre-ART) era (1987–1997, panel B), and the ART era (1997–2012, panel C) are shown. In addition, the characteristics of ART-naïve patients (D) and patients who received ART at the onset of lymphoma (E) in the ART era are shown. The numbers of cases are presented under each pie (see Table 1 for abbreviations).

**Figure 3:**
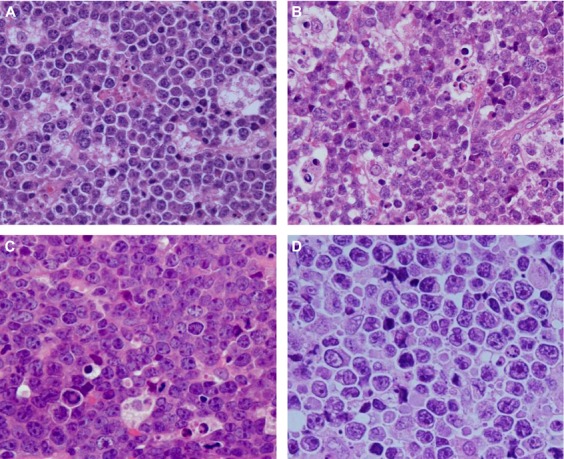
Differential diagnosis of diffuse large B-cell lymphoma and Burkitt lymphoma. (A) Burkitt lymphoma (BL). Each cell has a slight pleomorphism compared with a typical BL case. (B) BL. Although a starry sky pattern is shown, cells are large and pleomorphic. However, these cells are CD10^+^, CD20^+^, BCL-6^+^, and BCL-2^−^, with a Ki67 index of >90% and *MYC* rearrangement. (C) BL. The starry sky pattern is not clear, but some tingible body macrophages are observed. Cells have greater nuclear pleomorphism than those in typical BL. These cells are also CD10^+^, CD20^+^, BCL-6^+^, and BCL-2^−^, with a Ki67 index of >90% and *MYC* rearrangement, indicating the BL phenotype. (D) Diffuse large B-cell lymphoma (DLBCL) with *MYC* rearrangement. The cells in this case were CD10^+^, CD20^+^, BCL-6^+^, and BCL-2^−^, with *MYC* rearrangement and a Ki67 index of >90%. Extremely large cell morphology and severe nuclear pleomorphism without the starry sky pattern indicates DLBCL.

The clinical characteristics of the 207 ARL cases are summarized in Table 1 and Figure 2. The study group included 198 men and nine women, with a mean age of 45.4 years (range, 12–74 years). The HIV-1 transmission route was homosexual contact in 154 cases (74.4%) and heterosexual contact in 38 cases (18.4%). The remaining 10 patients (4.8%) were hemophiliacs and intravenous drug users. DLBCL was the predominant histological subtype diagnosed throughout the study period. Of all the DLBCL cases, 72 could be further subclassified as GC and non-GC types (Table 2). Seventeen cases of DLBCL were of the GC type (23.6%), and this number was lower than that reported in a study conducted in the US 33. Of 40 DLBCL cases that were subjected to CD5 testing, including 29 non-GC-type cases, none were positive for CD5, suggesting that CD5-positive DLBCL was rare among patients with ARL 34. Two cases of GC-type DLBCL showed *MYC* rearrangement. However, these cases were clearly distinguished from BL because of their morphological features, which included extremely large cells with severe pleomorphism and no starry sky pattern (Fig. 3). BL was the second most common subtype. Approximately 40% of BL cases did not show the typical morphology of BL, that is, medium-sized cells and a starry sky pattern (Fig. 3). These cells were larger and showed greater nuclear pleomorphism. However, these cells were CD10^+^, CD20^+^, BCL-6^+^, and BCL-2^−^; had a Ki67 index of >90%; and showed *MYC* rearrangement. These cases, initially classified as atypical BL according to the third edition of the WHO classification, were now categorized as BL according to the fourth edition 24,35. All cases of KSHV positivity were detected in homosexual patients. Among the nine PEL cases, six were of solid lymphomas (extracavitary PEL), two were of effusion lymphomas alone, and one was of both effusion and solid lymphomas. The HL cases showed the following subtypes: five were of the mixed cellularity type, two were of the nodular sclerosing type, and one was of the lymphocyte-depleted type. The other rare types of lymphomas included extranodal NK/T-cell lymphoma, nasal-type (two cases); angioimmunoblastic T-cell lymphoma (two cases); anaplastic large-cell lymphoma (ALK-negative) (one case); peripheral T-cell lymphoma, not otherwise specified (one case); adult T-cell lymphoma (one case); follicular lymphoma (one case); undefined DLBCL or BL (one case); EBV-associated lymphoproliferative disorder (one case); and undefined disease (one case).

**Table 2:** Comparison of germinal center (GC) and non-GC types of DLBCL.

Factor	GC type	Non-GC type	*P* value
*n*	17	55	–
Age, years (mean) [median, range]	51 [52, 31–76]	45 [44, 26–68]	0.12 (MW)
Men (%)	16 (94)	51 (93)	0.73 (CY)
CD4 (mean) [median, range]	197 [175, 17–824]	69 [31, 0–444]	**<0.01 (MW)**
ART (+) at onset (%)	47	19	**0.046 (CY)**
EBV-positive (%)	18	82	**<0.01 (C)**
CNS involvement (%)	7	59	**<0.01 (C)**
LN involvement (%)	64	28	**<0.01 (C)**
BM involvement (%)	13	8	0.51 (C)
1-year survival rate (%)	82	43	**<0.01 (C)**

ART, antiretroviral therapy; BM, bone marrow;C, chi-square test; CNS, central nervous system; CY, chi-square test with Yates' correction; DLBCL, diffuse large B-cell lymphoma;GC, germinal center; EBV, Epstein–Barr virus; LN, lymph node; MW, Mann–Whitney test

*P* values were calculated using the chi-square test (C), chi-square test with Yates' correction (CY), and Mann–Whitney test (MW). *P* values < 0.05 are presented in bold.

### CD4 counts and prognosis of each lymphoma subtype

CD4 counts differed among the lymphoma subtypes (Table 1). The mean CD4 counts of patients with BL and HL (249 and 235 cells/*μ*L, respectively) were significantly higher than those of patients with DLBCL, PBL, and PEL (86, 85, and 75 cells/*μ*L, respectively; *P* < 0.05, Mann–Whitney test). CD4 counts were also associated with EBV positivity in lymphomas. The mean CD4 count in patients with EBV-positive lymphoma (83.3 cells/*μ*L) was significantly lower than that in patients with EBV-negative lymphoma (246.2 cells/*μ*L; *P* < 0.01, Mann–Whitney test). Even among the patients with DLBCL, the mean CD4 count in patients with EBV-positive DLBCL (45.7 cells/*μ*L) was lower than that in patients with EBV-negative DLBCL (182.6 cells/*μ*L; *P* < 0.01, Mann–Whitney test). During the entire study period, 60.8% (66/121) of patients with EBV-positive lymphomas and 42.6% (31/79) of patients with EBV-negative lymphomas died within the first year of diagnosis; this indicated a better prognosis for EBV-negative cases than for EBV-positive cases, including HL cases (*P* < 0.001, chi-square test). However, in the ART era (after 1997), the survival rates of EBV-positive and EBV-negative cases of non-Hodgkin lymphoma were 56.9% (41/72) and 63.0% (46/73), respectively, and this difference was not significant (*P* = 0.455, chi-square test).

### Effect of ART on the onset of ARL

In total, 54 patients with ARL received ART at the onset of lymphoma. To determine whether ART introduction affected the onset of lymphoma, the clinicopathological characteristics of ARL occurring in patients receiving ART were compared with those occurring in ART-naïve patients (Fig. 2B–E, and Table 3). The 1-year survival rate was 65% in patients on ART, which was greater than that in ART-naïve patients (45%) (*P* = 0.012, chi-square test), suggesting a better prognosis for patients receiving ART than for ART-naïve patients. The histological differences between cases in the pre-ART era (1987–1997) and those in the ART era (1997–2012) are shown in Figure 2B and C. In the pre-ART era, 84% of ARL cases were of DLBCL, whereas the incidence of BL increased and the incidence of DLBCL decreased in the ART era. Considering the use of ART, the frequencies of BL and DLBCL did not differ significantly between patients receiving ART and ART-naïve patients (Table 3). However, the GC type of DLBCL was observed more frequently in patients receiving ART than in ART-naïve patients (Table 3). In addition, HL was observed more frequently in patients receiving ART (11.1%) than in ART-naïve patients (1.4%) during the entire study period. EBV positivity decreased and the 1-year survival rate was significantly improved in patients on ART. In addition, we analyzed 157 cases in the ART era to reveal the effect of ART in patients receiving this treatment (Fig. 2D and E). The incidence of HL increased from 1.0% in ART-naïve patients to 11.5% in patients receiving ART, even in the ART era (*P* < 0.01, chi-square test with Yates' correction). However, no significant differences were found in EBV positivity and LN involvement between patients receiving ART and ART-naïve patients in the ART era (Appendix Table 5).

**Table 3:** Effect of ART on the onset of AIDS-related lymphoma.

Factors	ART (−)	ART (+)	*P* value
*n*	146	53	—
Histology			
DLBCL	78 (53.4%)	23 (42.6%)	0.211(C)
(non-GC/GC)	(43/9)	(10/8)	**0.046 (CY)**
BL	36 (24.7%)	18 (33.3%)	0.192 (C)
PBL	13 (8.9%)	3 (5.6%)	0.653 (CY)
PEL	8 (5.5%)	1 (1.9%)	0.489 (CY)
HL	2 (1.4%)	6 (11.1%)	**<0.01 (CY)**
LBL-KSHV-MCD	0 (0%)	1 (1.9%)	0.596 (CY)
Other	9 (6.2%)	2 (3.7%)	0.763 (CY)
Age, years (mean) [median, range]	44 [42, 12–76]	49 [49, 29–75]	**<0.01 (MW)**
Men (%)	96	96	0.848 (CY)
CD4 (mean) [median, range]	104 [50, 0–560]	269 [176, 4–2431]	**<0.01 (MW)**
EBV-positive (%)	66	44	**0.010 (C)**
CNS involvement (%)	35	22	0.132 (C)
LN involvement (%)	61	58	0.701 (C)
BM involvement (%)	27	35	0.311 (C)
1-year survival rate (%)	45	65	**0.012 (C)**

ART, antiretroviral therapy; DLBCL, diffuse large B-cell lymphoma; GC, germinal center; BL, Burkitt lymphoma; PBL, plasmablastic lymphoma; PEL, primary effusion lymphoma; HL, Hodgkin lymphoma; LBL-KSHV-MCD, large B-cell lymphoma arising in Kaposi sarcoma-associated herpes virus-related multicentric Castleman disease; EBV, Epstein–Barr virus; CNS, central nervous system; LN, lymph node; BM, bone marrow; C, chi-square test; CY, chi-square test with Yates' correction; MW, Mann–Whitney test.

ART (−): patients who did not receive ART at the onset of lymphoma; ART (+): patients who received ART at the onset of lymphoma. *P* values < 0.05 are presented in bold.

### Correlation of biological markers with the prognosis of ARLs

The correlation of certain biological markers with the prognosis of ARL was also investigated (Table 4). The cases positive for two markers of GC-type B cells, CD10 and BCL-6, showed a significantly higher 1-year survival rate than did those negative for CD10 and BCL-6. However, the expression of CD20, CD138, BCL-2, IRF4/MUM1, or CD30 did not correlate with the 1-year survival rate. Infection with EBV and/or KSHV significantly reduced the 1-year survival rate. Thus, in ARL, the following conditions were associated with a poor prognosis: CD10 negativity, BCL-6 negativity, EBV positivity, and KSHV positivity.

**Table 4:** Prognostic significance of biological markers.

		1-year survival			
Markers	Result	Live	Death	Survival rate (%)	*P* value
CD20	+	77	78	49.7	0.863
	−	22	21	51.2	
CD10	+	51	22	69.9	**<0.01**
	−	32	44	42.1	
BCL-6	+	50	24	67.6	**<0.01**
	−	29	40	42.0	
CD138	+	11	8	57.9	0.659
	−	40	23	63.5	
BCL-2	+	30	18	62.5	0.986
	−	52	31	62.7	
IRF4/MUM1	+	32	30	51.6	0.778
	−	25	21	54.3	
CD30	+	29	20	59.2	0.931
	−	36	24	60.0	
EBER	+	49	66	42.6	**<0.01**
	−	48	31	60.8	
KSHV	+	3	8	27.3	0.034^*^
	−	50	26	65.8	

EBER, Epstein–Barr virus encoded small RNAs; KSHV, Kaposi sarcoma-associated herpesvirus.

*P*-values were calculated using the chi-square test or chi-square test with Yates' correction (^*^). *P* values < 0.05 are presented in bold.

## Discussion

In this study, we classified Japanese ARL cases according to histopathology. To the best of our knowledge, this is the first report to classify lymphomas histologically in a large number of HIV-1-infected patients according to the fourth edition of the WHO classification of lymphomas. Throughout the study period, DLBCL was the predominant histological subtype of ARL, followed by BL. EBV infection was present in 60% of all ARL cases. Although receipt of ART at the onset of lymphoma improved the 1-year survival rate of ARL, ART induction also resulted in an increase in the frequency of HL. In Figure 1, we present a flowchart for the diagnosis of ARL; the presence/absence of CD10, BCL-6, KSHV-LANA-1, and EBER were correlated with the prognosis of patients with ARL. This information will help in the early diagnosis of lymphomas in patients with AIDS.

Among HIV-1-infected patients, BL was sometimes composed of larger cells, compared to the typically observed medium-sized cells with plasmacytoid differentiation, and did not show the typical starry sky pattern. Such cases with atypical morphology were positive for CD10, BCL-6, and *MYC* rearrangement. As microarray studies demonstrated that these cases with atypical morphology shared a gene expression profile with BL 36,37, cases defined as atypical BL according to the third edition of the WHO classification were then categorized as BL according to the fourth edition, suggesting that the morphological spectrum of BL is very wide 24,35. In the present study, such atypical BL cases with typical BL phenotypes and atypical morphology were categorized as BL according to the fourth edition of the WHO classification 24. Although the morphology of atypical BL varied among cases, approximately 40% of BL cases in the present study showed atypical morphology, suggesting that atypical BL is frequent among AIDS patients with BL. DLBCL with *MYC* rearrangement should be distinguished from atypical BL. Two cases of lymphoma with CD20, CD10, and BCL-6 positivity and the *MYC* rearrangement with large cell morphology did not show any histological features of BL. Therefore, we categorized these cases as GC-type DLBCL with *MYC* rearrangement. Intermediate DLBCL/BL was newly defined in the fourth edition of the WHO classification 25. However, intermediate DLBCL/BL is a temporary category for high-grade B-cell lymphomas with a poor clinical outcome and is used mainly for cases with double- and triple-hit translocations 38. In addition, the fourth edition of WHO classification criteria did not include intermediate DLBCL/BL as an ARL 22. We did not encounter any cases of intermediate DLBCL/BL in the present study. However, as we could not perform a full chromosome analysis in all cases of BL and DLBCL, some cases in the present study might be categorized into this group. Further studies including a complete chromosome analysis will be required to clarify the presence of intermediate DLBCL/BL in ARL cases.

In the present study, we identified certain effects of ART introduction at the onset of lymphoma on the clinicopathological characteristics of ARL. An increasing number of HL cases in patients receiving ART have been reported in the United States and Japan 6,18,39. The mean CD4 count in patients with HL was higher than that in patients with other lymphomas, implying that the frequency of HL is associated with ART and immune reconstitution syndrome in HIV-1-infected patients. The proportion of BL cases did not increase significantly in patients on ART compared with ART-naïve patients (Table 3 and Fig. 2). As we reported in 2006, BL and PBL cases increased in the ART era compared with the pre-ART era 17. The present study also showed that the incidence of DLBCL drastically decreased from 84% among patients in the pre-ART era to 42% among ART-naïve patients in the ART era, whereas the BL incidence increased from 7% in the pre-ART era to 31% in the ART era (Fig. 2B and D). However, the frequencies of these subtypes did not differ significantly between patients receiving ART and ART-naïve patients in the ART era (Fig. 2D and E), suggesting that these changes were not associated with the introduction of ART. In the present study, the mean CD4 count of ART-naïve patients in the pre-ART era (62.2 cells/*μ*L) was significantly lower than that of ART-naïve patients in the ART era (121.5 cells/*μ*L; *P* < 0.01, Mann–Whitney test). Since 1998, the importance of HIV-1 testing is well recognized in the general population of Japan, and many persons with risk factors have visited clinics for HIV-1 testing, resulting in earlier detection of HIV-1 in the ART era than in the pre-ART era. Such early detection of HIV-1 in patients might be associated with histological differences between patients in the pre-ART era and the ART era. In contrast, the introduction of ART was associated with lower EBV positivity and higher 1-year survival rates in patients receiving ART than in ART-naïve patients.

Despite the increasing number of HL cases after the introduction of ART, DLBCL is still the most frequent subtype of ARL. In Japan, 23.6% of DLBCL cases were of the GC type. This rate is lower than that reported in the United States 33. Non-GC-type DLBCL was more frequent among HIV-1-negative cases in the United States. 32. In the present study, non-GC-type DLBCL showed higher rates of EBV infection, poorer prognosis, and more frequent CNS involvement than the GC type. In addition, the GC/non-GC ratio was higher in patients receiving ART than in ART-naïve patients. EBV infection was found to be less frequent among GC-type cases, and this may be associated with the high CD4 count among GC-type cases. Although a large clinical study reported no significant difference between the survival rates of patients with GC-type and non-GC-type DLBCL in the United States 33, another study showed that the non-GC type was associated with a poorer outcome 28. To estimate the prognosis of these groups, further clinical studies on a large set of DLBCL cases are warranted.

A diagnostic flowchart for ARL diagnosis, as used in the present study (Fig. 1), will be useful for the routine pathological diagnosis of ARL. We propose the use of CD3, CD20, CD10, BCL-6, IRF4/MUM1, BCL-2, Ki67, EBER, KSHV-LANA-1, CD138, CD30, and CD15 as markers for the classification of ARL. This set of biological markers can help distinguish between DLBCL, BL, PEL, HL, PBL, and T-cell lymphomas. GC-type DLBCL can also be distinguished from non-GC-type DLBCL by using this set of markers. *MYC* rearrangement should be analyzed to confirm the diagnoses of BL and DLBCL. Immunohistochemistry of CD20 is important for the differential diagnoses of PBL and KSHV-associated lymphomas because these lymphomas are usually negative for CD20 and have a poor prognosis. Statistical analyses identified CD10 negativity, BCL-6 negativity, EBV positivity, and KSHV positivity as risk factors for poor prognosis. The CD10 negativity, BCL-6 negativity, and EBV positivity may be associated with the poor prognosis of non-GC-type DLBCL, while the KSHV positivity was associated with the extremely low survival rates of 11 patients with KSHV-associated lymphoma (Table 1). EBV-positive cases had a poorer prognosis than did EBV-negative cases; however, EBV positivity was reduced with ART introduction. As DLBCL and BL are groups with biological heterogeneity and are not disease entities caused by a single genetic alteration, information regarding associated biomarkers may help predict their clinical outcomes.

## Appendix

**Table A1:** Effect of ART on the onset of ARL in the ART era.

Factors	ART (−)	ART (+)	*P*
n	105	52	–
Histology			
DLBCL	44 (41.9%)	21 (40.4%)	0.856 (C)
(non-GC/GC)	(26/9)	(10/8)	0.1665 (C)
BL	33 (31.4%)	18 (34.6%)	0.688 (C)
PBL	13 (12.4%)	3 (5.8%)	0.197 (C)
PEL	5 (4.8%)	1 (1.9%)	0.667 (CY)
HL	1 (1.0%)	6 (11.5%)	**<0.01 (CY)**
LBL-KSHV-MCD	0 (0%)	1 (1.9%)	0.719 (CY)
Other	9 (8.6%)	2 (3.8%)	0.448 (C)
Age, years (mean) [median, range]	45.1 [42.6, 25–76]	49.2 [48.5, 30–75]	**<0.01 (MW)**
Men (%)	97	96	0.880 (CY)
CD4 (mean) [median, range]	121.5 [76, 0–560]	280 [184, 6–2431]	**<0.01 (MW)**
EBV-positive (%)	59	44	0.076 (C)
CNS involvement (%)	20	20	0.981 (C)
LN involvement (%)	49	59	0.275 (C)
BM involvement (%)	30	35	0.556 (C)
1-year survival rate (%)	60	66	0.504 (C)

157 cases in the ART era (1997-) were analyzed. *P* values were calculated using the Chi-square test (C), Chi-square test with Yates correction (CY), and Mann–Whitney test (MW). See Table 1 for abbreviations. *P* values < 0.05 are presented in bold.
